# CD36 Signaling in Diabetic Cardiomyopathy

**DOI:** 10.14336/AD.2020.1217

**Published:** 2021-06-01

**Authors:** Xudong Zhang, Jiahui Fan, Huaping Li, Chen Chen, Yan Wang

**Affiliations:** Division of Cardiology, Tongji Hospital, Tongji Medical College and Hubei Key Laboratory of Genetics and Molecular Mechanisms of Cardiologic Disorders, Huazhong University of Science and Technology, Wuhan, China

**Keywords:** CD36, cardiomyocyte, endothelial cell, diabetic cardiomyopathy

## Abstract

Cluster of differentiation 36 (CD36), also referred to as scavenger receptor B2, has been shown to serve multiple functions in lipid metabolism, inflammatory signaling, oxidative stress, and energy reprogramming. As a scavenger receptor, CD36 interacts with various ligands, such as oxidized low-density lipoprotein (oxLDL), thrombospondin 1 (TSP-1), and fatty acid (FA), thereby activating specific downstream signaling pathways. Cardiac CD36 is mostly expressed on the surface of cardiomyocytes and endothelial cells. The pathophysiological process of diabetic cardiomyopathy (DCM) encompasses diverse metabolic abnormalities, such as enhanced transfer of cardiac myocyte sarcolemmal FA, increased levels of advanced glycation end-products, elevation in oxidative stress, impaired insulin signaling cascade, disturbance in calcium handling, and microvascular rarefaction which are closely related to CD36 signaling. This review presents a summary of the CD36 signaling pathway that acts mainly as a long-chain FA transporter in cardiac myocytes and functions as a receptor to bind to numerous ligands in endothelial cells. Finally, we summarize the recent basic research and clinical findings regarding CD36 signaling in DCM, suggesting a promising strategy to treat this condition.

## 1. Introduction

Cluster of differentiation 36 (CD36) was first reported as glycoprotein IV in 1977, detected as the fourth obvious band upon SDS-polyacrylamide protein gel electrophoresis of human platelet membranes [1]. It was subsequently demonstrated to be a macrophage receptor that mediated the uptake of oxidized LDL (oxLDL) and is involved in the phagocytosis of cells. Therefore, it is also called as the scavenger receptor [2]. As a transmembrane glycoprotein, CD36 is commonly expressed on the surface of specific cell types, including monocytes/ macrophages, endothelial cells (ECs), platelets, cardiomyocytes (CMs), skeletal myocytes, adipocytes, and some epithelial cells [3]. Moreover, CD36 could also be present in the endosomes, the endoplasmic reticulum, and the mitochondria and might shuttle between these subcellular organelles through vesicular transport to regulate lipid homeostasis and energy balance [4]. Furthermore, CD36 possesses many ligands according to specific cell types and triggers corresponding downstream signaling pathways to modulate lipid transfer [5], inflammatory cascade [6] and energy stability [7].

Diabetic cardiomyopathy (DCM) is defined as the presence of abnormal myocardial structure and function in patients with diabetes mellitus (DM), with the exclusion of hypertension, coronary artery disease, and other pre-existing cardiovascular diseases [8]. According to a report by the World Health Organization (WHO), 3.4 million individuals died of DM- associated complications in 2004; strikingly, this number is expected to double by 2030 [9]. Cardiovascular diseases including DCM and ischemic heart disease are major causes of death in both type 1 DM (T1DM) and type 2 DM (T2DM) [10]. In light of recent studies, by adjusting other cardiovascular risk factors, each 1% elevation of glycated hemoglobin A1c (HbA1c) levels was associated with a 30% increase in the risk of heart failure (HF) in T1DM [11]. Likewise, each 1% increase in HbA1c levels would result in an 8% rise in the risk of HF in T2DM [12].

Cardiac lipotoxicity and insulin resistance are typical abnormalities in DCM [13]. Moreover, uptake of circulating free fatty acids (FFAs) by the myocardium is mainly mediated by CD36 [14]. In addition, CD36-deficient patients presented a dramatic reduction in myocardial uptake of long-chain fatty acids (LCFAs), whereas^ 18^F-fluorodeoxyglucose (FDG) was increased in the myocardium [15, 16]. These metabolic changes were in accordance with the changes observed in CD36 knockout mice [17].

Our group mainly focused on the functions of microRNAs (miRs) in the pathophysiology of DCM. We found that miR-30c ameliorated cardiac metabolism disorders in DCM via peroxisome proliferator-activated receptor-gamma coactivator-1 beta (PGC-1β)/peroxisome proliferator-activated receptor alpha (PPARα) signaling [18]. Moreover, miR-21 improved DM-induced diastolic dysfunction by suppressing gelsolin [19]. Recently, we found that nuclear miR-320 triggered the transcription of fatty acid metabolic genes, including CD36, to cause DCM [20].

In this review, we first describe the structure of CD36 and focus on the roles of CD36 in signaling in CMs and ECs. We then summarize the effects of CD36 in various pathophysiological processes and the current CD36-related therapeutic strategies in DCM.

## 2. Different roles of CD36 in specific cell types

### 2.1. CD36 protein structure

**Figure 1.**
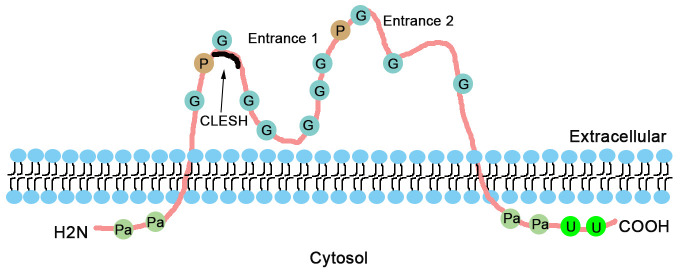
CD36 protein structure. The structure of CD36 on the cell membrane is represented with diverse post-translational modifications and ligand binding regions. CD36 has two transmembrane domains. Pa, palmitoylation; U, ubiquitylation; P, phosphorylation; G, glycosylation; CLESH, CD36 LIMP-II Emp sequence homology.

The structure of CD36 protein is shown in Figure 1. It is a pattern recognition receptor of approximately 88 kDa, and possesses two transmembrane domains, including a large extracellular region and two short cytoplasmic tails [21]. The extracellular loop has a large hydrophobic pocket called entrance 1, which is considered as a transport tunnel for major ligands, such as oxLDL and advanced glycation end products (AGEs) [22]. Furthermore, according to the crystal structure analysis, CD36 might possess another entrance (entrance 2) for FA transport [23]. In addition, thrombospondin type 1 (TSP1), one of the ligands of CD36, binds to CD36 via an extracellular region called the CD36 LIMP-II Emp sequence homology (CLESH) domain [24].

### 2.2 Transcriptional regulation of CD36

The transcription of CD36 is widely regulated by the PPAR transcription factor family that comprises of three tissue-specific distribution isoforms including PPARα, PPARβ/δ, and PPARγ [25]. PPARs are considered to directly modulate the transcription of target genes by binding to the so-called PPAR response element (PPRE) sequences in the promoter regions of genes [18]. Interestingly, several functional PPREs adjacent to the transcription start site (TSS) have been validated in the human and mouse CD36 gene [26, 27]. PPARα is mostly expressed in CMs to enhance the FA uptake via transcriptional activation of CD36 [28]. Moreover, adipose tissue-enriched PPARγ could reduce circulating FFA levels by increasing CD36-mediated FA transport in T2DM [29]. In addition, long-term fasting induces FA uptake by enhancing PPARγ binding to the CD36 promoter in human cardiac microvessel ECs [30]. PPARβ/δ plays a crucial role by regulating FA metabolism in muscles in response to exercise and fasting [31, 32]. Recently, the transcriptional factor forkhead box protein O1 (FoxO1) was shown to be upregulated in DCM and enhanced cardiac lipotoxicity by increasing CD36 transcription [33]. In addition, some miRNAs could regulate the transcriptional activation of CD36. Nuclear miR-320 positively regulates CD36 expression to exacerbate diabetes-induced cardiac dysfunction [20].

### 2.3 Translational regulation of CD36

Several molecules including proteins and miRNAs have been found to regulate CD36 translation. Y-box protein 1 mitigated oxLDL- mediated macrophage lipid uptake by suppressing CD36 protein levels rather than its transcription [34]. Moreover, in diabetic patients, high glucose levels enhanced the translational efficiency of CD36 and increased its cell surface expression in macrophages [35]. In addition, miR-200b-3p decreased cardiomyocyte apoptosis in DCM by inhibiting CD36 translation [36].

### 2.4 Post-translational regulation of CD36

A few post-translational modification events, including glycosylation, phosphorylation, ubiquitylation, and palmitoylation, play significant roles in CD36 trafficking and function.

The extracellular domain of CD36 has 10 potential glycosylation sites, most of which facilitate protein folding and trafficking without influencing ligand binding [37]. After translation, CD36 is extensively N-linked glycosylated at asparagine residues (Asn) by glycosyltransferases [37, 38]. Additionally, NADPH oxidase-4 (Nox-4) enhances fatty acid utilization and alleviates cardiac dysfunction in pressure overload-induced heart failure by increasing the O-linked N-acetylglucosamine (O-GlcNAcylation) of CD36 [39]. Moreover, glycosylation is a key process involved in the folding and stability of the CD36 protein [40]. It has been suggested that the glycosylation at Asn102 of CD36 is related to decreased intake of FA in spontaneously hypertensive rats [41].

Two phosphorylation sites including threonine-92 (Thr92) and serine-237 (Ser237) are located in the extracellular region which are recognized by protein kinase C (PKC) or PKA, respectively [42, 43]. Phosphorylation of CD36 *in vitro* at Thr92 inhibits binding to TSP-1 [44]. Furthermore, under flow conditions, phosphorylation at Thr92 affects the adherence of *Plasmodium falciparum*-infected erythrocytes to human microvascular endothelial cells [45]. Moreover, phosphorylation of CD36 at Ser237 is involved in palmitate uptake in human platelets [46]. Nevertheless, further *in vivo* studies are needed to be performed to validate the *in vitro* data.

In addition, four palmitoylation sites are separately located on the C- and N-terminal tails to adhere to the plasma membrane [47]. Suppression of CD36 palmitoylation alleviates lipid accumulation in mice with non-alcoholic steatohepatitis by triggering the adenosine 5’-monophosphate (AMP)-activated protein kinase (AMPK) pathway [48]. Acyl protein thioesterase 1 (APT1), one of the depalmitoylating enzymes, can depalmitoylate CD36 by recruiting tyrosine kinase SYK to induce the uptake of FAs and assist in weight gain in high-fat-diet (HFD)-fed mice [49]. Furthermore, despite insulin stress and AMPK activation, mutation in one of these four sites can impede CD36 translocation from the endosomes to the plasma membrane, indicating that palmitoylation is indispensable for the cycling of CD36 [50]. However, palmitoylation of CD36 in cardiac diseases has not been studied.

Two ubiquitination sites are located at the C-terminal tail. Insulin reduced CD36 ubiquitination whereas oleic acid treatment increased the ubiquitination of CD36, indicating that CD36 contributes to abnormal FA uptake during insulin-resistant state [51]. Additionally, Parkin, an E3 ubiquitin ligase, stabilizes CD36 on the plasma membrane in mice fed with high-fat and -cholesterol diet by monoubiquitination of CD36, which is contrary to the common fact that ubiquitinated proteins are degraded by the proteasome [52]. Similarly, the ubiquitin proteasome system 14 (UPS14) suppresses CD36 degradation in macrophages by cleaving its polyubiquitin chains and promoting foam cell formation [53]. However, the ubiquitination of CD36 in DCM has not been reported. In multiple cell types, CD36 can mediate various pathophysiological responses such as FA transfer and oxidative stress, via binding with different ligands. Next, we summarize the roles of CD36 in the main cell types involved in DCM.

## 3. CD36 in CMs

In CMs, CD36 mainly functions as a transporter to assist in the uptake of LCFAs. Indeed, CD36 contributes to nearly 70% of FA uptake in isolated rat CMs [54]. Moreover, it was found that the extracellular domain of CD36 coordinates with plasma membrane-localized fatty acid-binding protein (FABPpm) to facilitate cellular FA uptake [3]. Further, the intracellular region of CD36 provides a docking site for cytoplasmic FABP (FABPc), which causes the desorption of LCFAs from the membrane and transports them into the cytosol [55]. Then, most LCFAs are imported into the mitochondria and oxidized to release ATP for cellular utilization. Subsequently, the FA metabolites also increase which mostly consist of triacylglycerols (TAGs) stored in lipid droplets. Simultaneously, some of the metabolites are stored in diacylglycerols (DAGs) and ceramides (CERs) [56]. FAs, especially LCFAs, are considered as natural ligands of PPARs, and CM-enriched PPARα can directly enhance CD36 transcription [57]. In this process, CD36 positively regulates its own *de novo* synthesis by facilitating the entry and utilization of FAs.

In addition, nearly 50% of CD36 is stored in the endosomes in CMs [58]. Furthermore, CD36 plays a pivotal role in regulating the FA uptake rate via recycling between the endosomes and the plasma membrane. In the normal state, two stimulation factors such as insulin stress and muscle contraction can mediate the translocation of CD36 from the endosome to the sarcolemma [58, 59]. However, the signaling pathways activated by these two factors are different. The classical insulin receptor substrate 1 (IRS1)/phosphatidylinositol 3-kinase (PI3K)/protein kinase B (also called AKT) pathway is triggered upon the stimulation of CMs by insulin [60]. Then, the activated AKT inactivates the Akt substrate 160 (AS160) via Ser/Thr phosphorylation, followed by reduced downstream inhibition of Rab GTPase-activating proteins (Rabs). On the other hand, although increased muscle contraction upregulated intracellular AMPK, it restrained the expression of Tre-2/BUB2/cdc1 domain family 1 (TBC1D1), thereby diminishing the suppression of Rabs [61]. Subsequently, the activation of Rabs triggered the suppression of CD36 vesicular tra?cking.

CD36 tra?cking involves the participation of various vesicle-associated membrane protein (VAMP) families [62]. Moreover, the subcellular localization of CD36 in the endosomes is dependent on its intraluminal acidification, controlled by the proton pump vacuolar-type H^+^-ATPase (v-ATPase) [63]. Three VAMPs have been identified to be involved in CD36 tra?cking via systematic inhibition of diverse VAMPs in HL-1 cells [62]. VAMP4 is indispensable for CD36 transport between the endosomes and the intermediary vesicles. VAMP2 is the only protein that mediates CD36 translocation away from the sarcolemma. Regarding the transport from the intermediary vesicles to the sarcolemma, both VAMP2 and VAMP3 mediate this process. However, VAMP2 is regulated by AKT, a downstream target of the insulin signaling pathway, while VAMP3 is controlled by activation of AMPK signaling [7].

However, excessive lipids also disturb the normal CD36 subcellular recycling, thus inducing a permanent relocation of CD36 from the endosomes to the sarcolemma [7, 60]. Alkalinization of endosomes might account for the translocation of CD36 during excessive cardiomyocyte lipid supply [63]. Before the onset of insulin resistance, increased levels of intracellular LCFA induce the disassembly of v-ATPase cytosolic subcomplex V1 from the integral membrane V0 complex, thereby enhancing the relocation of CD36 to the sarcolemma [63]. In addition, lipid overload causes an increase in subsequent metabolites, including TAGs, DAGs, and CERs. The latter two metabolites suppress the insulin signaling pathway and lead to insulin resistance in distinct ways [3, 64, 65]. First, DAGs are considered as significant second messengers in the modulation of insulin signaling. DAGs can activate PKCs to inactivate IRS proteins via phosphorylation of their serine/threonine residues including serine residues such as Ser301 [65], Ser302 [66], Ser307 [67] and Ser1101 [68]. Second, CERs can directly suppress the activation of AKT by acting on its upstream protein phosphatase [69]. Moreover, inhibition of AKT leads to a decrease in VAMP2, thus suppressing the release of CD36 by the sarcolemma [62]. Furthermore, CERs may also activate PKC to compromise ATP generation and cell viability [70]. In addition, DAGs and CERs can inhibit NO production and increase the activity of NADPH oxidase, thereby yielding reactive oxygen species (ROS) by targeting PKC [71, 72]. Subsequently, the increase in ROS can also enhance CD36 expression via p53-mediated transcriptional regulation [73]. Moreover, suppression of insulin signaling impedes the translocation of glucose transport 4 (GLUT4) to the sarcolemma resulting in decreased glucose uptake [74]. All of these lead to an increase in FA uptake via a vicious feed-forward cycle of CD36 and decreased glucose uptake in CMs, hence disturbing the homeostasis of energy metabolism.

**Figure 2.**
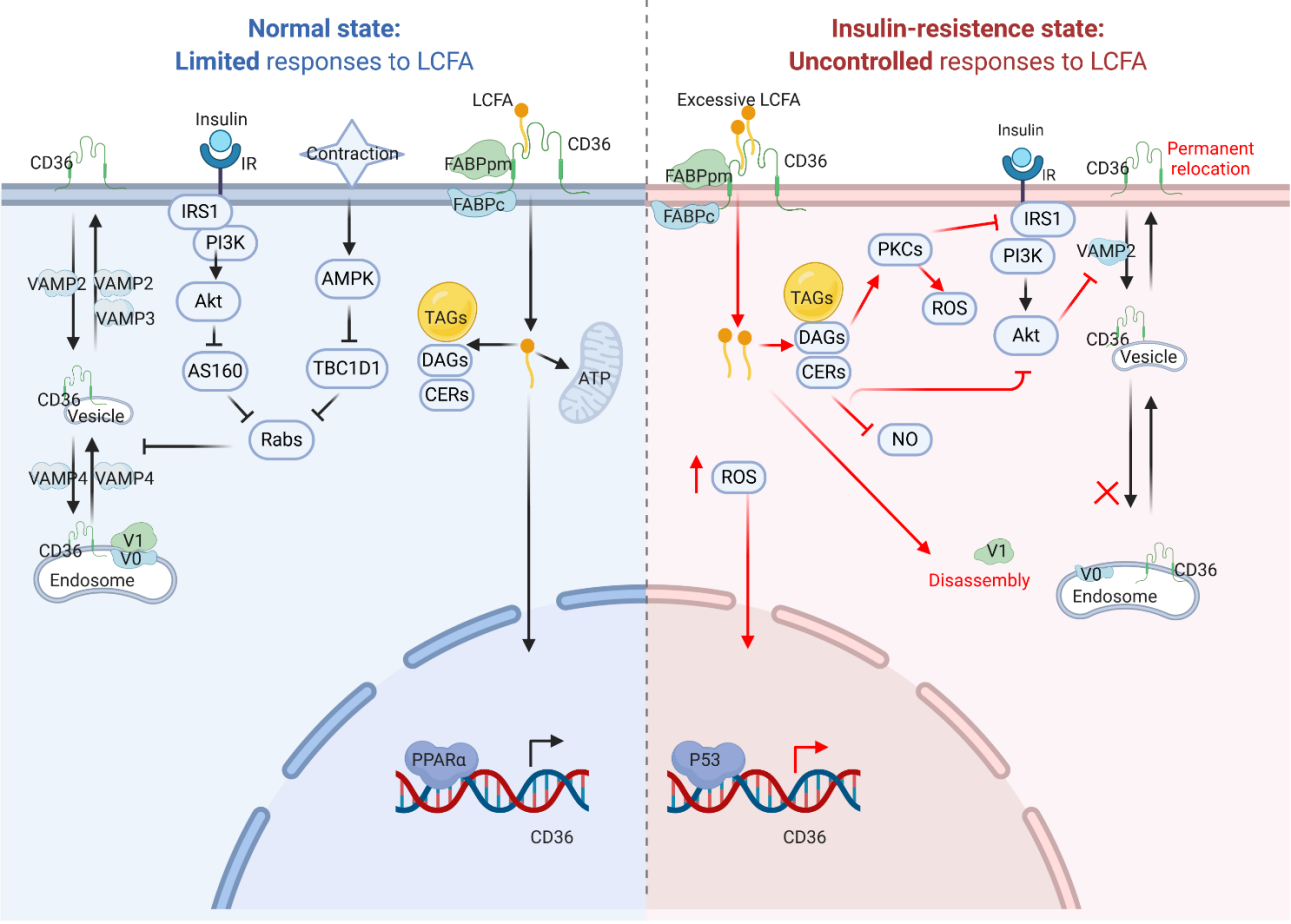
CD36 signaling pathway in cardiomyocytes. Under normal conditions (left), CD36 transfers LCFAs into cardiomyocytes, and LCFAs can promote CD36 transcription via PPARα binding to PPRE. Moreover, CD36 recycling occurs between endosomes and sarcolemma upon physiological stress, such as insulin stress and muscle contraction. During conditions of lipids oversupply (right), increased CD36 activity can enhance LCFA uptake to cause insulin resistance, endosome-sarcolemma recycling abnormalities and increased ROS production. LCFA, long-chain fatty acid; FABPpm, plasma membrane-localized fatty acid binding protein; FABPc, cytoplasmic FABP; TAG, triacylglycerol; DAG, diacylglycerol; Cer, ceramide; FAO, fatty acid oxidation; PPARα, peroxisome proliferator-activated receptor alpha; PPRE, PPAR response element; TSS, transcription start site; AMPK, adenosine 5’-monophosphate (AMP)-activated protein kinase; TBC1D1, Tre-2/BUB2/cdc1 domain family 1; Rabs, Rab GTPase-activating proteins; IRS1, insulin receptor substrate 1; PI3K, phosphatidylinositol 3-kinase; AKT, protein kinase B; AS160, Akt substrate 160; GLUT4, translocation of glucose transport 4; PKC, protein kinase C; V1, v-ATPase sub-complex V1; V0, v-ATPase sub-complex V0; VAMP, vesicle-associated membrane protein.

Collectively, CD36 acts as an LCFA transporter and regulates energy homeostasis in CMs (Fig. 2).

## 4. CD36 in ECs

**Figure 3.**
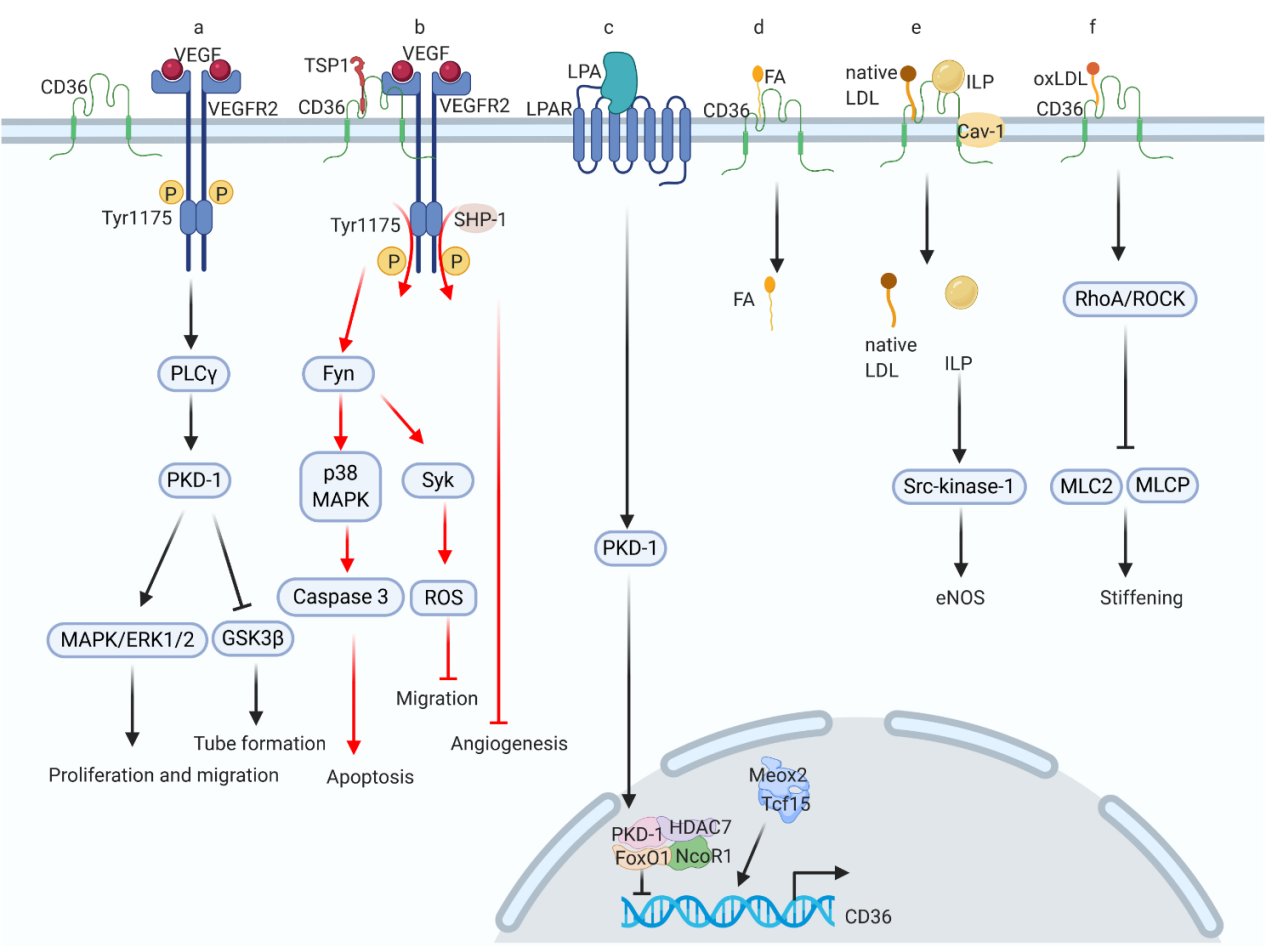
CD36 signaling pathways in endothelial cells. There are approximately seven signaling events related to CD36 in endothelial cells. (a) VEGF binds to its receptor to promote vascular migration and angiogenesis; (b) TSP-1 targets CD36 and CD36 forms a complex with VEGFR to inhibit VEGF signaling, thus promoting apoptosis and suppressing cell migration; (c) LPA can interact with its receptor and trigger downstream signaling to inhibit CD36 transcription; (d) CD36 transfers FA; (e) CD36 forms a complex with Cav-1 to mediate endocytosis of native LDL and intralipid to produce eNOS; (f) CD36 binds to oxLDL and mediates oxLDL-induced endothelial stiffening. VEGF, vascular endothelial growth factor; VEGFR2, vascular endothelial growth factor receptor 2; Tyr, tyrosine; PLC γ, phospholipase C γ, PKD-1, protein kinase D-1; MAPK, mitogen-activated protein kinase; ERK1/2, extracellular regulated protein kinase 1/2; GSK-3β, glycogen synthase kinase-3 beta; TSP-1, thrombospondin-1; SHP-1, srchomology 2 domain containing protein tyrosine phosphatase-1; Fyn, Src family kinase Fyn; Syk, tyrosine-protein kinase Syk; LPA, lysophosphatidic acid; GPCR, G-protein coupled receptor; LPAR, LPA receptor; HDAC7, histone deacetylase 7; FoxO1, forkhead box protein O1; NCoR1, nuclear receptor corepressor 1; Meox2, homeobox protein MOX-2; Tcf15, transcription factor 15; Cav-1, caveolin-1; eNOS, endothelial nitric oxide synthase; RhoA/ROCK, RhoA /Rho kinase; MLCP, myosin light-chain phosphatase; MLC2, myosin light-chain 2.

Mounting evidence suggests that CD36 acts as a key player in the physiological and pathophysiological functions of ECs. RNA sequencing analysis of isolated ECs from adult mouse hearts showed higher activation of the CD36 signaling pathway than the whole heart [75]. Likewise, single-cell RNA sequencing of normal mouse aorta indicated that CD36 can serve as a marker to differentiate the heterogeneity in EC populations [76]. The regulatory patterns of CD36 in ECs are diverse (Fig. 3). First, CD36 can be a negative modulator of angiogenesis, functioning as a receptor for TSP-1, since it contains the thrombospondin type I structural homology domain (TSR) [77]. CD36 related anti-angiogenesis process is involved in the vascular endothelial growth factor receptor (VEGFR) signaling cascade. When the ligand TSP-1 is not bound to CD36, VEGF can bind to its receptor, mainly VEGFR2 and induce phosphorylation at tyrosine (Tyr) 1175 site. As a result, phospholipase C γ (PLC γ)/protein kinase D-1 (PKD-1) signaling is activated [78]. Moreover, phosphorylation of PKD-1 triggers the mitogen-activated protein kinase (MAPK)/extracellular regulated protein kinase 1/2 (ERK 1/2) signaling cascade, and induces EC proliferation and migration [79]. On the other hand, PKD-1 also suppresses the activation of glycogen synthase kinase-3 beta (GSK-3β) via phosphorylation of its S9 residue and promotes tube formation [80].

Nevertheless, upon TSP-1 binding to CD36, the CD36-VEGFR2 complex is formed. Next, via the activation of the Src family kinases such as Fyn and p38 MAPK, the CD36-mediated pro-apoptotic pathway is triggered [81]. Furthermore, after the formation of the CD36-VEGFR2 complex, the Src homology two domain-containing protein tyrosine phosphatase-1 (SHP-1) is recruited to VEGFR2, which then mediates the dephosphorylation of Tyr 1175, thus inhibiting angiogenesis [81, 82]. Moreover, the activation of Fyn upregulates the expression of tyrosine-protein kinase Syk, which represses EC migration by upregulating NADPH oxidase and inducing the production of ROS [83].

Furthermore, lysophosphatidic acid (LPA), a ligand of the G protein-coupled LPA receptor (LPAR), can activate the PKD-1 signaling pathway, which is a key player in the VEGF signaling cascade [84]. Simultaneously, LPA signaling resulted in nuclear accumulation of PKD-1 and histone deacetylase 7 (HDAC7), where they formed a complex with FoxO1 and nuclear receptor corepressor 1 (NCoR1) to inhibit CD36 transcription, thus exhibiting a pro-angiogenic effect [85].

ECs function as a barrier for parenchymal cells, such as CMs, and prevent circulating FA uptake. However, CD36 mediates FA transport into ECs. The process of FA transfer into ECs could be induced by the formation of homeobox protein MOX-2 (Meox2) and transcription factor 15 (Tcf15) heterodimers in the nucleus [86]. However, using nanoscale secondary ion mass spectrometry (NanoSIMS) visualization technology, another group found that ^2^H-labeled lipids could still be transported into CMs via CD36-deficient ECs [87].

Intriguingly, recent studies have suggested that CD36 interacts with caveolin-1 (Cav-1) and is involved in endocytosis of native LDL and intralipid (ILP) [88, 89]. Moreover, ILP absorbed by ECs results in the upregulation of Src-kinase-1 and stimulates the production of endothelial nitric oxide synthase (eNOS) and NO release [89]. Therefore, this supports the clinical use of ILP to protect organs from ROS-mediated injuries. Additionally, ECs play an essential role in the pathogenesis of atherosclerosis. While this topic is not the major focus of this review, in summary, oxLDL enters ECs with the aid of CD36 and triggers the RhoA/Rho kinase (ROCK) cascade, which suppresses the phosphorylation of myosin light chain 2 (MLC2) and myosin light-chain phosphatase (MLCP) and causes EC stiffening [90]. Furthermore, the disturbed flow of atherosclerotic plaques also facilitates CD36-mediated uptake of oxLDL and increases EC stiffness [91, 92]. Overall, CD36 functions as a negative modulator of angiogenesis and a transporter of various lipids in ECs.

## 5. CD36 in the development of DCM

By performing ?uorescence-activated cell sorting to analyze the myocardial cell population, it was found that the adult murine heart is composed of approximately 56% cardiomyocytes, 27% cardiac fibroblasts, and the remainder includes ECs and vascular smooth muscle cells (VSMCs) [93]. Currently, the role of CD36 in cardiac fibroblasts remains unclear. In contrast, liraglutide, an analog of glucagon-like peptide-1 (GLP-1), attenuates high-glucose-induced proliferation of human myocardial fibroblasts via activation of the CD36/Jun N-terminal kinase (JNK)/activator protein-1 (AP-1) pathway [94]. In VSMCs, CD36 in response to oxLDL downregulates nuclear factor Nrf2, which then increases ROS accumulation in atherosclerosis [95].

Patients diagnosed with DCM at an early stage are usually asymptomatic [13]. However, metabolic abnormalities, including enhanced myocardial FA uptake and impaired insulin signaling cascade, are already initiated in these patients [96, 97]. Under physiological circumstances, FA metabolism and glucose metabolism are maintained in CMs [98]. During the progression of DM, there is a sharp loss (T1DM) or gradual reduction (T2DM) in insulin secretion, causing an increase in circulating glucose levels and more dependence on fatty acid oxidation [99, 100]. In addition, lipolysis of adipose tissues increases the circulating levels of FAs and enhances the capacity of CD36 to transport FAs into CMs [101]. CD36-deficient mice exhibited increased cardiomyocyte TAG accumulation and cardiac dysfunction after high-fat diet feeding compared with the controls [102]. Recently, applying proton (^1^H)- magnetic resonance spectroscopy (MRS) technology, it was found that myocardial TAGs of diabetic patients were between 1.5- and 2.3-fold compared to non-diabetic controls [103-105]. The levels of TAGs predicted concentric left ventricular (LV) remodeling and subclinical contractile dysfunction [105]. Moreover, FA metabolites, DAGs and CERs suppressed PI3K/AKT insulin signaling to induce insulin resistance and triggered PKC to generate mitochondrial ROS, which caused CM apoptosis and myocardial necrosis [106, 107]. Excessive mitochondrial ROS production could also boost cardiac fibrosis via the transforming growth factor β1 (TGF-β1)/p38 MAPK signaling cascade [108]. Interestingly, CD36 selectively recognizes mature, mitochondria-rich human pluripotent stem cell (hPSC)-derived CMs [109]. Moreover, mitochondrial dysfunction plays a crucial role in the progression of insulin resistance and lipid overload in mice [110, 111]. Increased FAs in CMs excessively activated PPARα expression and enhanced the transcription of CD36 (Fig. 2). Elevated PPARα levels led to reduced activity of the Ca^2+^ pump in the sarcoplasmic reticulum and impaired excitation-contraction coupling of CMs [112].

In addition to PPARα, PPARγ also plays a significant role in regulating CD36 expression in DCM. PPARγ activation could improve insulin sensitivity by inducing adiponectin and lowering FFA levels via boosting CD36-mediated FFA transport to adipose tissue, thereby reducing the lipotoxicity of the myocardium. Therefore, PPARγ activation is being extensively used as a therapeutic strategy to treat T2DM [113-115]. However, cardiac-specific overexpression of activator protein 1 member JunD aggravated HFD-induced cardiac dysfunction and lipid accumulation via JunD/PPARγ/CD36 signaling [116]. Additionally, PPARγ CM-specific transgenic mice showed increased CD36 expression, enhanced lipid stores, and impaired cardiac function [117]. The difference between global PPARγ agonists and CM-specific PPARγ activation may be due to difference in tissue distribution of PPAR isoforms. Therefore, the cardiac function of patients should be evaluated when PPARγ agonists are used to treat DM. Recently, CD36 was also reported to regulate PPARγ signaling in DCM. MiR-200b-3p alleviated cardiocyte apoptosis by inhibiting the CD36/PPARγ signaling pathway in a HFD-induced DCM model [36]. Furthermore, growth hormone-releasing peptides could function as ligands to target CD36 and lead to downstream activation of PPARγ in metabolic diseases [25]. However, PPARγ agonists have not yet been recommended to treat DCM.

These aforementioned pathological changes are consistent with the progression of DCM, which starts with the onset of myocardial fibrosis and related diastolic dysfunction, followed by cardiac systolic dysfunction, ultimately leading to clinical heart failure [118].

In addition, DCM is usually accompanied by microvascular recruitment and rearrangement [119]. Reduced coronary flow reserve has been correlated with an increase in cardiac death independent of DM, suggesting that EC dysfunction might affect the prognosis of DCM [120]. Intriguingly, EC-specific CD36-KO mice rather than CM-specific CD36-KO mice exhibited diminished uptake of LCFAs into the heart, indicating that ECs played a key role in LCFA transport [121]. Meox2 and Tcf15 function as pivotal transcriptional regulators of ECs. However, mice with combined Meox2 and Tcf15 haplodeficiency presented diminished FA transfer to CMs due to downregulated expression of CD36 in ECs [86]. Increased expression of cardiac TSP-1 in DCM, an important ligand of CD36 in ECs, induced microvascular rarefaction [122]. Moreover, CD36 is predominantly present in small blood vessels rather than in large arteries in both human and mouse hearts, indicating that endothelial CD36 plays a crucial role in the metabolism of CMs [121]. Additionally, several studies have demonstrated that DAGs in ECs of diabetic rats remained at a high level for at least three weeks [123]. Moreover, high glucose levels enhanced oxLDL uptake through CD36 transporter in cultured microvascular endothelial cells and heart tissues of diabetic rats, thus inducing ROS production and cardiac injuries [124].

Some translational studies have been conducted on the therapeutic effects of CD36 signaling. For instance, GLP-1 receptor (GLP-1R) agonists are usually administered to patients with diabetes [125]. Our group has demonstrated that a GLP-1 analog can attenuate the lipotoxicity in DCM by inhibiting the activation of ROCK/PPARα/CD36 signaling [126]. Simultaneously, GLP-1 has been demonstrated to inhibit CD36 translocation during the initial stage of DCM by triggering the GLP-1R/AKT-dependent pathway [127]. Recently, sodium-glucose cotransporter-2 (SGLT-2) inhibitors, as antidiabetic drugs, have been suggested to lower the incidence of HF-related admission and mortality in patients with DM [128]. Moreover, empagliflozin, an SGLT-2 inhibitor, can reduce cardiotoxic lipids via CD36 and ameliorate autophagy in a rat model of T2DM [129]. In addition, some endogenously active small molecules may exert their effects on DCM via CD36. Hydrogen sulfide (H_2_S) can modulate VAMP3 ubiquitylation to block CD36 translocation and diminish droplet formation in the cardiac tissues of db/db mice [130]. DM-induced increase in CD36 expression can be suppressed by endogenous molecules such as fibroblast growth factor 21 (FGF21) [131] and a recently validated adipokine, apelin [132], thus lowering FA uptake. In addition, sulfo-N-succinimidyl oleate (SSO), a CD36 inhibitor, can improve metabolic disorders and cardiac dysfunction in rats with type 2 diabetes [56]. In 2-deoxyglucose-exposed mouse microvascular endothelial cells, metformin can robustly upregulate the expression of the CD36 ligand TSP1, and increase TSP1-CD36 co-localization, thereby causing a marked reduction in phosphorylated VEGFR2 and inhibition of angiogenesis [133]. Moreover, according to the German KORA F4 population study, circulating protein levels of TSP-1 were positively associated with pre-diabetic morbidity [134].

Overall, CD36 in CMs and ECs is involved in the pathophysiological processes of DCM, and CD36 signaling-related treatments are promising therapeutic strategies for DCM.

## 6. Conclusions and future perspectives

CD36, a well-known FA transporter, plays an essential role in the regulation of metabolism during DCM. In addition, CD36 can bind to diverse ligands to trigger different signaling pathways during disease progression. In this review, we first described the basic structure of the CD36 protein and the CD36 regulation at transcriptional, translational and post-translational level. Second, we discussed the involvement of CD36 signaling in the pathophysiological processes of CMs and ECs. Finally, we summarized the function of CD36 in DCM and discussed the therapeutic potential of targeting CD36 signaling.

However, in addition to CMs and ECs, CD36 is also expressed in SMCs, platelets, and macrophages, which are involved in the development of various cardiovascular diseases [135-137]. DCM is a complex syndrome, including a myriad of disorders, such as abnormal mitophagy, impaired Ca^2+^ handling, and increased AGEs [118]. This review presents the significant role of CD36 in the pathogenesis of DCM.

Cell surface molecules are essential for cellular communication and can be regarded as molecular fingerprints [138]. The CD nomenclature was initially used for leukocyte surface antigen classification and has been recognized as a universal system for classification. Moreover, the CD code indicates the general molecular binding partner of antibodies (glycoprotein, glycan) and the CD number represents the chronological order [139]. The CD system can be classified as glycans and lectins, which reflect the potential mode of molecular recognition in cell biology [140]. The CD name of a glycan can indicate changes in the structure of proteins and polysaccharides, enabling adjustment to specific environments, such as CD17, that regulates enzyme activities during neutrophil migration [138, 141]. In addition, information regarding cognate lectins is finely regulated to translate into cellular effects, such as CD1d, which is involved in T cell-mediated protective reactions against microbial pathogens [142]. Among the members of the CD family, CD36 was initially identified as a glycoprotein in platelet membranes [3].

Due to the insidious symptoms and scarce effective treatment options for DCM, we would like to focus on several encouraging targets based on CD36. Uncontrolled recycling of CD36 between the endosomes and the sarcolemma can be inhibited by targeting the v-ATPase of endosomes and VAMPs. Second, cardiac PPARα, the major upstream molecule of the CD36 gene, may be a promising potent target. In addition, insulin resistance may be improved by reducing the accumulation of TAGs and CERs in the body. Moreover, CD36 of ECs can be inhibited to reduce cardiac FA uptake and mitigate DM-induced cardiac dysfunction. In addition, TSP-1, a CD36 ligand in ECs, may be associated with diabetes-related microvascular rearrangement. Furthermore, reducing CD36-mediated oxidative stress has been validated to diminish DM-induced cardiomyocyte apoptosis.

Nevertheless, CD36 is essential for sufficient ATP generation when hypertrophic cardiomyocytes are not substantially affected by increased levels of FFAs [143]. During this circumstance, due to limited FA supply, loss of CD36 aggravates cardiac dysfunction in pressure overload-induced HF [144, 145]. Likewise, the increased expression of ROS-generating Nox-4 in the pressure-stressed heart could drive the O-GlcNAcylation of CD36, which balances the energy metabolism and improves adverse cardiac remodeling [39]. In conclusion, both enhanced and reduced FA metabolism may be detrimental to cardiac function.

Taken together, CD36 plays a pivotal role in the progression of DCM. CD36 signaling may be a promising therapeutic target for DCM.
